# Efficiently sparse listing of classes of optimal cophylogeny reconciliations

**DOI:** 10.1186/s13015-022-00206-y

**Published:** 2022-02-15

**Authors:** Yishu Wang, Arnaud Mary, Marie-France Sagot, Blerina Sinaimeri

**Affiliations:** 1 Université de Lyon, Université Lyon 1, CNRS, Laboratoire de Biométrie et Biologie Evolutive UMR 5558, 69622 Villeurbanne, France; 2 ERABLE team, Inria Grenoble Rhône-Alpes, Villeurbanne, France; 3 Luiss University, Rome, Italy

**Keywords:** Cophylogeny, Enumeration, Equivalence relation, Dynamic programming

## Abstract

**Background:**

Cophylogeny reconciliation is a powerful method for analyzing host-parasite (or host-symbiont) co-evolution. It models co-evolution as an optimization problem where the set of all optimal solutions may represent different biological scenarios which thus need to be analyzed separately. Despite the significant research done in the area, few approaches have addressed the problem of helping the biologist deal with the often huge space of optimal solutions.

**Results:**

In this paper, we propose a new approach to tackle this problem. We introduce three different criteria under which two solutions may be considered biologically equivalent, and then we propose polynomial-delay algorithms that enumerate *only* one representative per equivalence class (without listing all the solutions).

**Conclusions:**

Our results are of both theoretical and practical importance. Indeed, as shown by the experiments, we are able to significantly reduce the space of optimal solutions while still maintaining important biological information about the whole space.

## Background

Reconstructing the evolutionary history of parasites (or symbionts) and their hosts has many applications such as for example identifying and tracing the origins of emerging infectious diseases [1–3]. These studies have become increasingly more important with the large amount of publicly available sequence data. A powerful framework for modeling host-parasite co-evolution is provided by *cophylogeny* models which derive evolutionary scenarios for both hosts and parasites (usually evolutionary trees are computed from DNA sequence data). Co-evolution is usually modeled as a problem of mapping the phylogenetic tree of the parasites to the one of the hosts (see e.g. [4–7]). Such mapping, called a reconciliation, allows the identification of some biologically motivated events: (a) cospeciation, when the parasite diverges in correspondence to the divergence of a host species; (b) duplication, when the parasite diverges but not the host; (c) host-switching, when a parasite switches from one host species to another independent of any host divergence; and (d) loss, which can describe for instance speciation of the host species independently of the parasite, which then follows just one of the new host species. Finding the “best” reconciliation is modeled as an optimization problem by assigning a cost to each of the different types of events and then seeking the reconciliations that minimize the total cost (computed in an additive way). In practice, there may often be many optimal solutions which, although having the same total cost, can be quite different among them and correspond to different biological scenarios. Most of the software proposed in the literature therefore do not rely only on one optimal solution but enumerate *all* of them (e.g. [6–10]). A crucial issue is that often the number of optimal solutions is unrealistically large (exponential in the size of the trees) [6, 11–14], making it practically impossible to analyze each one of them separately.

To tackle this problem, we observe that although many of the solutions can be indeed very different, a large number of them are quite similar and can be considered biologically equivalent. We thus first propose various equivalence relations for grouping the reconciliations that may be considered biologically equivalent, then we provide algorithms which efficiently enumerate *only* the equivalence classes or one representative reconciliation per class.

### State of the art

Many methods have been proposed in the literature to deal with the large number of optimal reconciliations. Some early approaches propose sampling the space of optimal reconciliations uniformly at random [15, 16]. However, as the optimal reconciliation space can be both large and heterogeneous [17], this does not guarantee that important information is not lost.

Other methods try to understand the structure of the space of solutions by computing some global properties such as the frequency of the events across the space [16], the diameter of the space [17], the pairwise distance among the optimal reconciliations [18]. In a similar direction, other methods propose a single reconciliation (e.g. a “median” reconciliation) to represent the whole space of optimal ones [11, 14, 19]. However, the results presented in [12, 14, 17, 18] show that the space can be very diverse and making inferences from a single reconciliation might lead to conclusions that can be contradicted by other optimal reconciliations. The method in [19] has been generalized in [20] in order to find a set of *k* medoids, or *k* centers that represent the space. However, these algorithms have a running time of $O\,(n^{k+3} \log k)$ (where *k* is the number of clusters and *n* is the size of the trees) and are thus not applicable in practice. Finally, in [10, 13] the solutions are clustered using a similarity distance among the reconciliations. However, in some cases the results of the clustering can be hard to interpret (see Section “Experimental results”).

### Our contribution

In this paper, we propose an approach that is entirely different from the ones discussed in the state of the art section. We first formally define under what conditions two solutions can be considered biologically equivalent. Some first steps in this direction were done in [21] where two notions of equivalence were first considered. However, the method presented in [21] requires first the listing (i.e. the enumeration) of all the optimal solutions and then clustering them according to the equivalence notion.

Here we introduce three different equivalence relations. We then propose an algorithm that efficiently enumerates the set of “equivalence classes” or that enumerates one representative per class *without* having to first generate all of them. The algorithms that we present are *polynomial-delay*, meaning that the time between the output of any solution and the next one is bounded by a polynomial function of the input size. Our results are of both practical and theoretical importance. Indeed, the problem of enumerating equivalence classes, and particularly the generation of representative solutions is a challenge in the context of enumeration algorithm. It has been identified as a need in different areas, such as genome rearrangements [22], artificial intelligence [23], pattern matching [24, 25], or the study of RNA shapes [26].

It is worth mentioning that the theoretical results in this paper have inspired the introduction of a general framework to enumerate equivalence classes for a whole class of problems which can be addressed by dynamic programming algorithms [27].

## Model description

### Definitions

In this section, we formally present the phylogenetic tree reconciliation problem that was originally introduced by Goodman et al. in 1979 [28]. We start by providing some definitions that will be used in the paper.

For a directed graph *G*, we denote by *V* (*G*) and *A* (*G*) respectively the set of nodes and the set of arcs of *G*. The out-neighbors of a node *v* are called its children. We consider ordered rooted trees in which arcs are directed away from the root. For a tree *T*, we denote by *L* (*T*) the set of leaf nodes, i.e. those nodes without children, and denote by *r* (*T*) the root of *T*; the non-leaf nodes are called the internal nodes of *T*. A full rooted binary tree is a rooted tree in which every internal node has two children.

We denote by *p* (*w*) the parent of a node *w*. The children of a node *w* are denoted by a couple (i.e. an ordered pair) $\textsf {ch}(w)$. If there exists a directed path from a node *v* to a node *w*, the node *w* is called a *descendant* of *v*, and *v* is called an *ancestor* of *w*; if moreover $v\ne w$, we say that *w* is a *proper descendant* of *v*, and that *v* is a *proper ancestor* of *w*. If neither *w* is an ancestor of *v* nor *w* is an ancestor of *v*, we say that the two nodes are *incomparable*, and denote this as $v\not \sim w$. We denote by $\textsf {LCA}\,(v,w)$ the lowest common ancestor of two nodes *v* and *w*. The subtree of *T* rooted at a node *v* containing all descendants of *v* is denoted by $T|_v$. Finally, we denote by $d_T\,(v,w)$ the distance, i.e. the number of arcs on a directed path, between two comparable nodes *v* and *w* in *T*.

We define next the phylogenetic tree reconciliation problem (for short, the reconciliation problem). Let *H* and *P* be respectively the rooted phylogenetic trees of the host and parasite species, both binary and full. Let $\sigma$ be a function from *L* (*P*) to *L* (*H*),  representing the parasite/host associations between extant species. A reconciliation is a function $\phi$ that assigns, for each parasite node $p\in V (P)$, a host node $\phi (p)\in V(H)$, and satisfies the conditions stated in Definition 1. A reconciliation must induce an event function $E_\phi$ on *V*(*P*) which associates each parasite node *p* to an event $E_\phi (p)$. The set of events is denoted by ${\mathcal {E}} := \{{\mathbb {C}} ,\,{\mathbb {D}},\,{\mathbb {S}},\,{\mathbb {T}}\}$; the leaf parasite node has a special event ${\mathbb {T}}$; for internal parasite nodes, the event $E_\phi (p)$ is one among three options: *cospeciation* ${\mathbb {C}}$, *duplication* ${\mathbb {D}}$, and *host-switch* ${\mathbb {S}}$. The event for an internal node *p* will depend on the hosts that are assigned by $\phi$ to *p* and to the two children $p_1$ and $p_2$ of *p*. In Definition 1, this dependency is expressed by $E_\phi\, (p) := E\,(\phi \,(p),\phi \,(p_1),\phi\, (p_2))$.

#### Definition 1

(*Reconciliation, Event of a node*) Given two phylogenetic trees *H* and *P*, and a function $\sigma : L(P)\rightarrow L(H)$, a reconciliation of $(H, P, \sigma )$ is a function $\phi : V(P)\rightarrow V(H)$ satisfying the following:

1. For every leaf node $p\in L(P),$ $\phi (p)$ is equal to $\sigma (p)$, and $E_\phi (p)={\mathbb {T}}$.
2. For every internal node $p\in V(P)\setminus L(P)$ with children $(p_1,\,p_2),$ exactly one of the following applies:
  - $E\left( \phi (p),\phi (p_1),\phi (p_2)\right) ={\mathbb {S}}$, that is, either $\phi (p_1)\not \sim \phi (p)$ and $\phi (p_2)$ is a descendant of $\phi (p)$, or $\phi (p_2)\not \sim \phi (p)$ and $\phi (p_1)$ is a descendant of $\phi (p)$,
  - $E\left( \phi (p),\phi (p_1),\phi (p_2)\right) ={\mathbb {C}}$, that is, $\textsf {LCA}(\phi (p_1),\phi (p_2)) = \phi (p)$, and $\phi (p_1)\not \sim \phi (p_2)$,
  - $E\left( \phi (p),\phi (p_1),\phi (p_2)\right) ={\mathbb {D}},$ that is, $\phi (p_1)$ and $\phi (p_2)$ are descendants of $\phi (p)$, and the previous two cases do not apply.

In a reconciliation, an internal parasite node can be additionally associated to a number of *loss events*. The loss event is denoted by ${\mathbb {L}}$. A loss can only occur in conjunction with another event (${\mathbb {S}}$, ${\mathbb {C}}$, or ${\mathbb {D}}$), and the definition of the number of losses splits into several cases according to the accompanying event. We give in Definition 2 the number of loss events associated to an internal node *p*, called the *loss contribution* $\xi _\phi (p)$. Since the loss contribution is also determined by the hosts that are assigned to *p* and to the children of *p*, we will also write $\xi _\phi (p):=\xi (\phi (p),\phi (p_1),\phi (p_2))$.

#### Definition 2

(*Loss contribution*) Let $\phi :V(P)\rightarrow V(H)$ be a reconciliation. Let *p* be an internal node of the parasite tree with children $p_1,\,p_2.$ Its *loss contribution* $\xi _\phi (p)$ is defined by:

$$\begin{aligned} \xi _\phi (p) := {\left\{ \begin{array}{ll} d_H(\phi (p), \phi (p_1))&\quad\text {if}\,E_\phi (p)= {\mathbb {S}}\;\text {and}\;\phi (p)\not \sim \phi (p_2),\\ d_H(\phi (p), \phi (p_2))&\quad\text {if}\,E_\phi (p)= {\mathbb {S}}\;\text {and}\;\phi (p)\not \sim \phi (p_1),\\ d_H(\phi (p), \phi (p_1))+d_H(\phi (p),\phi (p_2))-2&\quad\text {if}\; E_\phi (p)={\mathbb {C}},\\ d_H(\phi (p), \phi (p_1)) + d_H(\phi (p),\phi (p_2))&\quad\text {otherwise,\;}E_\phi (p)={\mathbb {D}}. \end{array}\right. }\end{aligned}$$

The function $E_\phi$ partitions the set of internal parasite nodes into three disjoint subsets according to their event; these subsets are denoted by $V^{\mathbb {C}}(P), \,V^{\mathbb {D}}(P),\,V^{\mathbb {S}}(P)$. The number of occurrences of each of the three events together with the number of losses make up the *event vector* of the reconciliation $\phi$:

#### Definition 3

(*Event vector*) The *event vector* of a reconciliation $\phi$ is a vector of four integers consisting of the total number of each type of events ${\mathbb {C}},\,{\mathbb {D}},\,{\mathbb {S}},\,$ and ${\mathbb {L}}$, i.e.

1 $$\begin{aligned} \vec {e}\, (\phi ) := \left( \left| V^{\mathbb {C}}(P)\right| ,\,\left| V^{\mathbb {D}}(P)\right| ,\,\left| V^{\mathbb {S}}(P)\right| ,\,\sum _{p\,\in V(P)\setminus L(P)}\, \xi _\phi (p)\right) \,. \end{aligned}$$

Given a *cost vector* $\vec {c}:=\left( c({\mathbb {C}}),\,c({\mathbb {D}}),\,c({\mathbb {S}}),\,c({\mathbb {L}})\right)$ assigning a real number to each type of event, the *cost of a reconciliation* $\phi$ is equal to the dot product between the cost vector and the event vector $\text {cost}\,(\phi ) := \vec {c}\cdot \vec {e}\,(\phi )$. We are now ready to formulate the optimization version of the reconciliation problem: Given two phylogenetic trees *H* and *P*, a function $\sigma :L(P)\rightarrow L(H),$ and a cost vector $\vec {c}$, find a reconciliation $\phi$ of $(H, P, \sigma )$ of minimum cost.

**Fig. 1:**
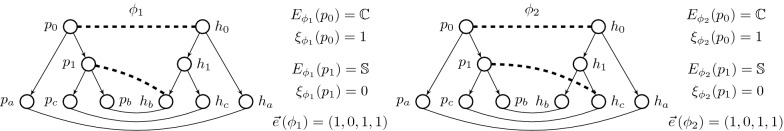
Example of two reconciliations $\phi _1$ and $\phi _2$ on the same input. For each reconciliation, we draw the parasite tree on the left, the host tree on the right; the solid edges represent the associations for the leaf parasite nodes; the dashed edges represent the associations for the internal parasite nodes

In Fig. 1, we show two different reconciliations on the same input $(H,P,\sigma )$. Depending on the cost vector, these reconciliations may or may not be optimal. Notice that if the cost vector is (0, 0, 0, 0), any valid reconciliation will be optimal.

### Dynamic programming algorithm

The reconciliation problem can be solved by dynamic programming. One of the first methods which took into account all the events described in the previous section was introduced by Michael Charleston in 1998 [29] and has been improved since by different authors. These methods have different ways of dealing with time feasibility which makes the problem hard on undated trees. We will not discuss this further in the present paper, except for mentioning that in the dynamic programming approach presented in this section, the trees are considered undated, and the time feasibility issue can be dealt with in a subsequent step as described in [6]. On the other hand, we show in this section a formulation of the dynamic programming algorithm in terms of a certain directed graph which we will define. The graph structure can be seen as a means for efficiently enumerating all optimal solutions of the optimization problem, and more importantly, we will use it later in Section “Algorithmic results” for enumerating equivalence classes of optimal reconciliations.

#### Recurrence relations

Given an instance $(H,P,\sigma , \vec {c})$, the minimum cost of a reconciliation can be found by dynamic programming. Recall that ${\mathcal {E}}:=\{{\mathbb {C}},\,{\mathbb {D}},\,{\mathbb {S}},\,{\mathbb {T}}\}$ is the set of possible events for a node. Let $U:= V\,(P)\times V\,(H)\times {\mathcal {E}}$. We call a triple $(p,h,e)\in U$ a *cell* of the dynamic programming table. Consider a function $f:U\rightarrow {\mathbb {R}}\cup \{\infty \}$, where the *value* of a cell *f* (*p*, *h*, *e*) is defined to be the minimum cost of a reconciliation between the subtree $P|_p$ (i.e., the subtree of *P* rooted at the node *p*) and the host tree *H* mapping *p* to *h*, such that the event of *p* is *e*. Then *f* can be computed as follows:

1. If *p* is a leaf,
  2 $$\begin{aligned} f(p,h,e) = {\left\{ \begin{array}{ll}0&{}\text {if}\;h=\sigma (p)\;\text {and}\;e={\mathbb {T}},\\ \infty &{} \text {otherwise}.\end{array}\right. }\end{aligned}$$
2. Otherwise, *p* is an internal node with children $(p_1,p_2).$ In this case,
  3 $$\begin{aligned} f(p,h,e) = \mathop {min}\limits _{\begin{array}{c} E(h,h_1,h_2)=e\\ h_1,h_2\in V(H)\\ e_1,e_2\in {\mathcal {E}} \end{array}} f({p_1}, {h_1},e_1) + f({p_2},{h_2},e_2) + c(e) + c({\mathbb {L}})\,\xi (h,h_1,h_2)\,. \end{aligned}$$

The minimum cost of a reconciliation is then given by $\min _{h\in V(H),e\in {\mathcal {E}}} f({r(P)}, h, e).$

#### ad-AND/OR graphs and solution subtrees

In order to find one optimal reconciliation or to efficiently enumerate all optimal reconciliations, a directed graph can be constructed from the recurrence relations Eqs. (2) and (3): it is a compact representation of all series of computations performed by dynamic programming which result in the optimal cost value. To do this, we rely on a well-known structure in Computer Science, that is the *AND/OR graph* [30]. More specifically, we consider a particular flavor of AND/OR graphs that we call *acyclic decomposable AND-OR graphs*. This structure is known for having an intimate relationship with dynamic programming on a tree.

##### Definition 4

(*ad-AND/OR graph*) A directed graph *G* is an *acyclic decomposable AND/OR graph* (an ad-AND/OR graph) if it satisfies the following:

- *G* is a DAG.
- *G* is bipartite: its node set *V*(*G*) can be partitioned into $({\mathcal {A}}, {\mathcal {O}})$ so that all arcs of *G* are between these two sets. Nodes in ${\mathcal {A}}$ are called *AND nodes*; nodes in ${\mathcal {O}}$ are called $\text {OR}^+$ *nodes*.
- Every AND node has in-degree at least one and out-degree at least one. The set of nodes with out-degree zero is then a subset of ${\mathcal {O}}$ and is called the set of *goal nodes*; the remaining $\text {OR}^+$ nodes are simply the *OR nodes*. The subset of OR nodes of in-degree zero is the set of *start nodes*.
- *G* is decomposable: for any AND node, the sets of nodes that are reachable from each one of its child nodes are pairwise disjoint.

##### Definition 5

(*Solution subtree*) A *solution subtree* *T* of an ad-AND/OR graph *G* is a subgraph of *G* which: (1) contains exactly one start node; (2) for any OR node in *T* it contains exactly one of its child nodes in *G*, and for any AND node in *T* it contains all its children in *G*.

The set of solution subtrees of *G* is denoted by ${\mathcal {T}}(G)$. It is immediate to see that a solution subtree is indeed a subtree of *G*: it is a rooted tree, the root of which is a start node. If we would drop the requirement of *G* being decomposable, the object defined in Definition 5 would not be guaranteed to be a tree.

##### Definition 6

(*Subgraph starting from a set of nodes*) Let *G* be an ad-AND/OR graph. Let ${\mathcal {O}}$ be a set of $\text {OR}^+$ nodes of *G*. The *subgraph of G* *starting from* ${\mathcal {O}}$, denoted by $G/{\mathcal {O}}$, is the subgraph obtained from *G* by setting ${\mathcal {O}}$ as the new set of start nodes (i.e. by removing all nodes that are not reachable from ${\mathcal {O}}$ through directed paths).

#### The reconciliation graph

The reconciliation graph is a concept already present in the literature [6, 16, 31]. Since, depending on the application, slightly different definitions of this structure exist, to avoid ambiguity, we describe how to construct the *reconciliation graph* of a given instance of the reconciliation problem from the recurrence Eqs. (2, 3).

The construction is done in two steps. In the first step, we build a graph in which every node retains an additional attribute, its *value*, and every $\text {OR}^+$ node is uniquely labeled by a dynamic programming cell $(p,h,e)\in U$. In the second step, we *prune* the graph by removing nodes that do not yield optimal values.

1. For each $(p,h,e)\in U$ such that *p* is a leaf, create a goal node labeled by (*p*, *h*, *e*); its value is equal to 0 if $h=\sigma (p)$ and $\infty$ otherwise. Then, for each $(p,h,e)\in U$ in the post-order of *V* (*P*), let $p_1,p_2$ be the two children of *p*,
  i. For each $(p_{1},h_{1},e_{1})$ and each $(p_{2},h_{2},e_{2})$ such that $E(h,h_1,h_2)=e$, create an AND node, connect it to the two $\text {OR}^+$ nodes respectively labeled by $(p_1,h_1,e_1)$ and $(p_2,h_2,e_2)$. Its value is equal to the sum of the values of its two children, plus $c\,(e)+c({\mathbb {L}})\,\xi \,(h,h_1,h_2)$.
  ii. Create a single OR node, connect it to every AND node created in the previous step. Its label is (*p*, *h*, *e*), and its value is the minimum of the values of its children.
2. For each $(r\,(P),h,e)\in U$, remove the OR node labeled by that cell unless its value is equal to the optimal cost. For each OR node *s*, remove the arc to its child AND node $s_i$ if the value of $s_i$ is not equal to the value of *s*. Finally, remove recursively all AND nodes without incoming arcs.

**Fig. 2:**
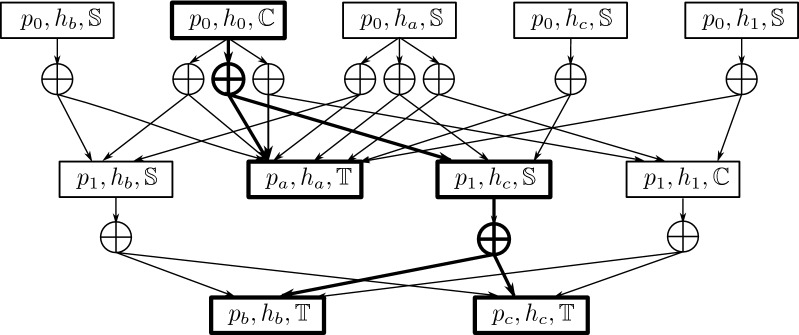
Example of a reconciliation graph for the input $(H,P,\sigma )$ in Fig. 1. Crossed circles are AND nodes. Rectangles are $\text {OR}^+$ nodes. The cells with which the $\text {OR}^+$ nodes are labeled are written inside. One solution subtree is shown in bold

It can be checked that the reconciliation graph is indeed an ad-AND/OR graph as defined in Definition 4. An $\text {OR}^+$ node labeled by (*p*, *h*, *e*) is a start node if and only if $p=r\,(P)$, and is a goal node if and only if $p\in L\,(P)$. It is also immediate to see that each AND node in the reconciliation graph has exactly one in-neighbor and exactly two children. We will consider the two children as a couple: for an AND node *s*, if its in-neighbor is labeled by (*p*, *h*, *e*) and its two children $s_1$ and $s_2$ are respectively labeled by $(p_1,h_1,e_1)$ and $(p_2,h_2,e_2)$, we will say that $s_1$ is the first child and $s_2$ is the second child of *s* if $p_1$ and $p_2$ are respectively the first and second child of *p*; otherwise, we say that $s_1$ is the second child and $s_2$ is the first child. Keeping the correct order of the children, we can extend the notation “$\textsf {ch}$” to the set of nodes of the reconciliation graph: if *s* is an AND node, $\textsf {ch}(s)$ is the couple (ordered pair) of the two child $\text {OR}^+$ nodes of *s*; if *s* is an OR node, $\textsf {ch}(s)$ is simply the set of its AND child nodes. For an OR node, we will typically be interested not in its children but in its set of “grandchildren”, hence we introduce here a new notation. If *s* is an OR node, we call the *grandchild couples*, denoted by $\textsf {gch}(s)$, the union of the children of its child AND nodes (it is a set of couples of $\text {OR}^+$ nodes): $\textsf {gch}(s):=\bigcup _{s_i\in \textsf {ch}(s)}\textsf {ch}(s_i)$. Notice that an $\text {OR}^+$ node can appear as grandchild of two different nodes, and can also appear in two different grandchild couples of a same node (see Fig. 2).

The dynamic programming algorithms for the reconciliation problem which enable the efficient enumeration of all optimal reconciliations are based on the following observation:

##### Proposition 7

*Let* $(H,P,\sigma ,\vec {c})$ *be a given instance of the* reconciliation problem. *The reconciliation graph* *G*, *constructed as described in the* *previous paragraph is an ad-AND/OR graph, and the set* ${\mathcal {T}}(G)$ *of solution subtrees of* *G* *correspond bijectively to the set of optimal reconciliations*.

To see this, consider an $\text {OR}^+$ node *s* labeled by a cell $(p,h,e)\in U$ of the dynamic programming table. For the subgraph $G/\{s\}$ (see Definition 6), the following can be proven by induction: the set of solution subtrees ${\mathcal {T}}(G/\{s\})$ corresponds bijectively to the set of optimal reconciliations of the dynamic programming subproblem at (*p*, *h*, *e*), i.e. the optimal reconciliations between the subtree *P*|*p* and *H* such that *p* is mapped to *h* and the event of *p* is *e*. In practice, to convert a solution subtree $T_1\in {\mathcal {T}}(G)$ into a reconciliation $\phi$, we only need to look at the labels (*p*, *h*, *e*) of the $\text {OR}^+$ nodes in $T_1$ (a reconciliation can simply be viewed as a collection of triples of the form (*p*, *h*, *e*)). We will henceforth use interchangeably the terms *solution subtrees* of the reconciliation graph and *optimal reconciliations* of the problem instance.

The reconciliation graph can be constructed using $O(|V\,(P)||V\,(H)|^3)$ time and space complexity [6]. After the construction, the total number of optimal reconciliations can also be computed. It is a well-known folklore result that the set of solution subtrees of an ad-AND/OR graph can be enumerated efficiently: the delay between outputting two consecutive solutions is linear in the size of the solution. Therefore, there is an algorithm with a $O(|V\,(P)||V\,(H)|^3)$ time pre-processing step and *O*(|*V*(*P*)|) time delay for enumerating the optimal reconciliations.

Figure 2 shows a reconciliation graph based on the same input $(H,P,\sigma )$ as in Fig. 1 with nine solution subtrees. Among these nine reconciliations, four have event vector (0, 0, 2, 0), two have (1, 0, 1, 0), two have (1, 0, 1, 1) ($\phi _1$ and $\phi _2$ of Fig. 1), and one has (2, 0, 0, 0). The event vector of the reconciliation shown in bold is (1, 0, 1, 1).

## Definitions of the equivalence relations

In this section, we first introduce four definitions of equivalence between reconciliations and study the relationship between them, then we explain the motivation for defining such equivalence relations and state the problems of enumerating the equivalence classes and counting the size of each class. The algorithmic contribution solving these problems and the experimental results will be presented in the subsequent sections.

### Definitions

In Definition 8, 9, 10, we give three equivalence relations on the set of optimal reconciliations. One is based on a global property, the event vector, which is already defined in Definition 3. The other two equivalence relations are based on “local properties”, i.e. on the event $E_\phi (p)$ and the host $\phi (p)$ that are assigned by $\phi$ for each parasite node *p*.

#### Definition 8

(*V-equivalence*) Two reconciliations $\phi _1$ and $\phi _2$ are *Vector-equivalent*, or for short *V-equivalent*, if their event vectors are equal: $\vec {e}\,(\phi _1) = \vec {e}\,(\phi _2)$.

#### Definition 9

(*E-equivalence*) Two reconciliations $\phi _1$ and $\phi _2$ are *Event-equivalent*, or for short *E-equivalent*, if $E_{\phi _1}(p) = E_{\phi _2}(p)$ for all $p\in V(P)$.

#### Definition 10

(*CD-equivalence*) Two reconciliations $\phi _1$ and $\phi _2$ are *Cospeciation-Duplication-equivalent*, or for short *CD-equivalent*, if $E_{\phi _1}(p) = E_{\phi _2}(p)$ for all $p \in V(P)$ (i.e. they are E-equivalent), and the hosts of non-host-switch parasite nodes are the same: $E_{\phi _1}(p)\ne {\mathbb {S}} \implies \phi _1(p) = \phi _2(p)$.

Each one of these equivalence relation splits the set of optimal reconciliations of a given instance into *equivalence classes*, i.e. subsets of pairwise equivalent reconciliations. One *representative* of an equivalence class is simply a reconciliation in the corresponding subset. We will abuse the terminology and call equivalence classes the objects that best represent the common property of the reconciliations in that subset. A reconciliation in a particular equivalence class will then be a reconciliation satisfying that property.

#### Definition 11

(*Equivalence classes*) In this paper, the term *equivalence class* has the following meanings, depending on the equivalence relation:

- For the V-equivalence relation, a *V-equivalence class* is an event vector $\vec {e}\,,$ i.e. a vector of four integers.
- For the E-equivalence relation, an *E-equivalence class* is a function $E:V(P)\rightarrow {\mathcal {E}}$ that associates each node of the parasite tree with an event.
- For the CD-equivalence relation, a *CD-equivalence class* is a function $E^{\text {CD}}:V(P)\rightarrow {\mathcal {E}}\times \left( V(H)\cup \{?\}\right)$ that associates each node of the parasite tree with an ordered pair (*e*, *h*), where either
  - *e* is an event between ${\mathbb {T}}$, ${\mathbb {C}}$ and ${\mathbb {D}}$ and *h* is a node of the host tree, or
  - *e* is the host-switch event ${\mathbb {S}}$ and *h* is a special symbol ?.

We can make the following remarks about the relationships between these equivalence relations. CD-equivalent reconciliations are also E-equivalent. Being E-equivalent implies that the first three elements of their event vectors are equal. As we only consider reconciliations having the same minimum cost, if the cost of a loss event $c({\mathbb {L}})$ is nonzero, E-equivalent reconciliations necessarily have the same number of losses, hence are also V-equivalent. On the other hand, if $c({\mathbb {L}}) =0$, E-equivalent reconciliations are not necessarily V-equivalent.

In Fig. 1, the pair $\phi _1$ and $\phi _2$ are equivalent under all three equivalence relations. In Fig. 2, the nine reconciliations split into four V-equivalence classes (the four event vectors).

### Motivation and challenges

The first and foremost motivation of defining equivalence relations is the need of capturing useful biological information from the set of optimal reconciliations, when this set is too large for manual analyses or for exhaustive enumeration. The V-equivalence classes already conveys some information about the co-evolutionary history of the hosts and their parasites. Indeed, a high number of cospeciations may indicate that hosts and parasites evolved together, while a high number of host-switches may indicate that the parasites are able to infect different host species. Under the scope of the E-equivalence relation, we are also interested in which parasites are associated to each type of event (disregarding losses).

The CD-equivalence relation is motivated by the idea that when a host-switch happens, there may be various hosts that can be selected as the parasite’s “landing site”. In this case, we choose to consider as equivalent those reconciliations for which, while the hosts that receive the switching parasites may differ, all the other parasite-host associations (not corresponding to a host-switch) are the same. These reconciliations are similar and often indistinguishable without additional biological information. Indeed, take the two reconciliations $\phi _1$ and $\phi _2$ in Fig. 1: they are identical except for one switching parasite $p_1$, which is mapped to $h_b$ by $\phi _1$ and to $h_c$ by $\phi _2$. Since $h_b$ and $h_c$ are two sibling nodes sharing the same parent in the host tree, without further information, there is no good way to tell apart the two reconciliations $\phi _1$ and $\phi _2$, hence we consider them as equivalent.

Equipped with our definitions of equivalence classes, we aim at studying the features of the set of optimal reconciliations by enumerating the equivalence classes. Naively, one would iterate through every reconciliation and record their properties, then report the equivalence classes, and, only at the end, report the statistics of the reconciliations in each equivalence class. However, when the number of reconciliations is too large, for example, $> 10^{42}$ (see Section “Experimental results” and [32]), the naive method is not feasible.

The challenge is then to enumerate directly the equivalence classes of optimal reconciliations without enumerating the latter explicitly. Concretely, the set of optimal reconciliations will be represented implicitly as ${\mathcal {T}}(G)$, the set of solution subtrees of a reconciliation graph *G*. Given a reconciliation graph as input, we will tackle the following problems:

- Count the number of equivalence classes.
- Enumerate the equivalence classes.
- Study a particular equivalence class. That is, given an equivalence class,
  - Count the number of reconciliations in that class,
  - Find one representative (i.e. one optimal reconciliation) of that class,
  - Enumerate all reconciliations of that class.

## Algorithmic results

### V-equivalence class enumeration

The enumeration of V-equivalence classes (i.e. all event vectors among the optimal reconciliations) can be achieved by a simple modification of the dynamic programming algorithm.

First, we can notice that the number of different event vectors is bounded by a polynomial. Let $n=|V(H)|$ and $m=|V(P)|$. The first three elements of any event vector necessarily sum up to $\frac{m-1}{2}$, the number of internal parasite nodes, hence there are only $O(m^2)$ possible combinations. The loss contribution $\xi _\phi (p)$ for each parasite node *p* for any $\phi$ is at most twice the diameter of the host tree (i.e. twice the maximum distance between two nodes), so the fourth element of any event vector is bounded by *O*(*nm*). Therefore, the number of event vectors is bounded by $O(nm^3)$.

We are interested in the following two problems: listing all event vectors, and, given a particular event vector, listing one (or all) optimal reconciliations of that event vector. Both can be done without much difficulty by doing some additional book-keeping in the dynamic programming algorithm, i.e. during the construction of the reconciliation graph. The idea is to remember the set of event vectors in every step, corresponding to the event vectors of the optimal solutions of the current dynamic programming subproblem. Then, for each event vector, one reconciliation (or all reconciliations) of the V-equivalence class can be found by backtracking.

Recall that if *s* is an $\text {OR}^+$ node of the reconciliation graph, the solution subtrees of the subgraph $G/\{s\}$ correspond to the optimal reconciliations of the dynamic programming subproblem identified by the cell (*p*, *h*, *e*) with which *s* is labeled. We now define the set $\textsf {EV}$ of an $\text {OR}^+$ node *s* to be the set of event vectors of ${\mathcal {T}}(G/ \{s\})$, that is the event vectors of the set of optimal reconciliations of the corresponding dynamic programming subproblem. Then, the sets $\textsf {EV}$ can be computed as follows (for simplicity, we will identify an $\text {OR}^+$ node with the cell (*p*, *h*, *e*) with which it is labeled):

- For each goal node $(p,h,{\mathbb {T}})$, $\textsf {EV}(p,h,{\mathbb {T}}) := \{(0,0,0,0\}$.
- For each OR node (*p*, *h*, *e*), let $\{\left( (p^i_1,h^i_1,e^i_1),(p^i_2,h^i_2,e^i_2)\right) \}_{1\le i\le k}$ be its set of grandchild couples, then $\textsf {EV}(p,h,e)$ can be computed as
  4 $$\begin{aligned} \bigcup _{1\le i\le k}\;\mathop {\bigcup }\limits _{\begin{array}{c} \vec {u}\in \textsf {EV}(p^i_1,h^i_1,e^i_1)\\ \vec {w}\in \textsf {EV}(p^i_2,h^i_2,e^i_2) \end{array}}\left\{ \vec {u}+\vec {w}+\left( 0,0,0,\xi (h,h_1,h_2)\right) + {\left\{ \begin{array}{ll} ( 1, 0,0,0)&\text {if}\;e=\mathbb {C} \\ ( 0, 1,0,0) &\text {if}\;e=\mathbb {D} \\ ( 0, 0,1,0) & \text {otherwise,}\;e=\mathbb {S} \end{array}\right. }\right\} \,. \end{aligned}$$

The set of event vectors of ${\mathcal {T}}(G)$ that we seek is the union $\bigcup _s \textsf {EV}(s)$ taken over the set of start nodes of *G*, i.e. the $\text {OR}^+$ nodes labeled with a cell of the form (*r*(*P*), *h*, *e*).

Overall, for each of the $O\,(n^3m)$ nodes of the reconciliation graph, we need to keep an extra set of size $O\,(nm^3)$. The space complexity is therefore $O\,(n^4m^4)$. For each OR node and for each of its $O\,(n^2)$ grandchild couples, we need to compute the Cartesian sum of two sets of $\textsf {EV}$s of size $O\,(nm^3)$ each; this can be done naively in time $O\,(n^2m^6)$ (to improve this, see, e.g. [33]). The overall time complexity is $O(n^5m^7)$.

The backtracking technique for finding one optimal reconciliation given its event vector is quite standard. Here we present it concisely without proof. We define a function backtrack that takes two parameters: an $\text {OR}^+$ node *s* in the reconciliation graph *G* and a vector $\vec {v}$ satisfying $\vec {v}\in \textsf {EV}(s)$. The function returns an optimal subproblem reconciliation $\phi _s\in {\mathcal {T}}(C/\{s\})$ such that $\vec {e}\,(\phi _s)=\vec {v}$. We choose to represent a reconciliation as a sequence of triples of the form (*p*, *h*, *e*). The function backtrack($s,\vec {v}$) can be implemented as follows:

1. Let (*p*, *h*, *e*) be the cell with which *s* is labeled. Output the triple (*p*, *h*, *e*). If *s* is a goal node, stop. Otherwise, go to Step 2.
2. Let $\{\left( (p^i_1,h^i_1,e^i_1),(p^i_2,h^i_2,e^i_2)\right) \}_{1\le i\le k}$ be the grandchild couples of *s*. Find any index *i* such that there exists $\vec {u}\in \textsf {EV}(p_1^i,h^i_1,e^i_1)$ and $\vec {w}\in \textsf {EV}(p_2^i,h^i_2,e^i_2)$ such that the sum inside the big braces of Eq. (4) is equal to $\vec {v}$ (such *i* necessarily exists). Choose any such $\vec {u}$ and $\vec {w}$. Then do backtrack($(p_1^i,h^i_1,e^i_1),\vec {u}$) and backtrack($(p_2^i,h^i_2,e^i_2),\vec {w}$).

Given a start node *s* and an event vector $\vec {v}\in \textsf {EV}(s)$, it suffices to call backtrack($s,\vec {v}$) to get one representative of the V-equivalence class $\vec {v}$. Finally, if we replace “any” by “all” in Step 2 of backtrack, we can easily adapt the algorithm in such a way that it enumerates all reconciliations, or counts the number of reconciliations of a V-equivalence class.

### E-equivalence class enumeration

By Definition 11, an E-equivalence class is a function from the set of nodes *V*(*P*) of the parasite tree to the set ${\mathcal {E}}:=\{{\mathbb {C}},{\mathbb {D}},{\mathbb {S}},{\mathbb {T}}\}$ of events. In this section, we will represent an E-equivalence class as a set *T* of ordered pairs of the form (*p*, *e*) where $p\in V(P)$ and $e\in {\mathcal {E}}$. In the same manner, a reconciliation $\phi$, i.e. a solution subtree in ${\mathcal {T}}(G)$, can be written as a set of ordered triples of the form (*p*, *h*, *e*). We say that a reconciliation $\phi$ *belongs to the E-equivalence class* *T*, and denote it as $\pi (\phi )=T$, if for each $(p,h,e)\in \phi$, there exists a unique couple $(p,e)\in T$. Using this notation, a set of couples of the form (*p*, *e*) is an E-equivalence class if and only if there exists $\phi \in {\mathcal {T}}(G)$ such that $\pi (\phi )=T$; the set of all E-equivalence classes is denoted by $\pi ({\mathcal {T}}(G))$.

The problem of studying a particular E-equivalence class is easy: given an E-equivalence class *T*, the reconciliation graph *G* can be pruned in such a way that its set of solution subtrees corresponds to the reconciliations that belong to the class *T* (we simply need to remove all OR nodes unless its label (*p*, *h*, *e*) corroborates the given class: $(p,e)\in T$). Counting and enumerating the E-equivalence classes are, however, more challenging problems. We will at present concentrate on the problem of enumerating all E-equivalence classes.

The algorithm is based on the simple idea of traversing the reconciliation graph in a top-down fashion (a similar approach can be used in the algorithm that finds all the solution subtrees). In order to obtain a polynomial time delay algorithm, during the traversal, we can no longer consider the nodes one by one; the sets of nodes that are in the solution subtrees of the same E-equivalence class must be traversed together. To make this clear, it is convenient to define the *color* of the $\text {OR}^+$ nodes; an E-equivalence class will then simply be a set of colors.

#### Definition 12

(*Color of a node, Color couple*) 

- If an $\text {OR}^+$ node *s* in the reconciliation graph is labeled by $(p,h,e)\in U$, we say that *s* is *colored by* the ordered pair $(p,e)\in V(P)\times {\mathcal {E}}$.
- Let $s_1$ and $s_2$ be two $\text {OR}^+$ nodes colored respectively by $(p_1,e_1)$ and by $(p_2,e_2)$. The *color couple* of the couple of nodes $(s_1,s_2)$ is the couple of colors $((p_1,e_1),\,(p_2,e_2))$.

To enumerate the E-equivalence classes by a top-down recursive traversal of the reconciliation graph, our algorithm should achieve the following goal: given a set ${\mathcal {O}}$ of $\text {OR}^+$ nodes of the same color (*p*, *e*), enumerate $\pi ({\mathcal {T}}(G/{\mathcal {O}}))$, i.e. all E-equivalence classes of the subgraph $G/{\mathcal {O}}$. Any such a class will include the color (*p*, *e*). If *p* is not a leaf, the events of the two children of the node *p* are given by the color couples of the grandchild couples $\textsf {gch}\,({\mathcal {O}})$ (by extension, $\textsf {gch}$ of a set of nodes is the union of $\textsf {gch}$ of every node in the set). A naive algorithm can be described as follows: for each color couple $((p_1,e_1),\,(p_2,e_2))$ of $\textsf {gch}\,({\mathcal {O}})$, first take the union ${\mathcal {O}}_1$ of the first grandchildren of color $(p_1,e_1)$ and the union ${\mathcal {O}}_2$ of the second grandchildren of color $(p_2,e_2)$, then call the algorithm on ${\mathcal {O}}_1$ and independently on ${\mathcal {O}}_2$, and finally combine the results together, that is, perform a Cartesian product between $\pi ({\mathcal {T}}\,(G/{\mathcal {O}}_1))$ and $\pi ({\mathcal {T}}\,(G/{\mathcal {O}}_2))$.

The pitfall of the naive approach is that not every combination between the E-equivalence classes of the reconciliations of the two child subtrees is valid. Our algorithm, shown in Algorithm 1, can be viewed as an improved version of the naive algorithm in which particular care has been taken to ensure that only valid combinations are outputted. Along with each E-equivalence class *T*, it also outputs a set $\widetilde{{\mathcal {O}}}$ which is a subset of the input set ${\mathcal {O}}$: it is equal the union of the root $\text {OR}^+$ nodes of all solution subtrees $\phi \in {\mathcal {T}}(G/{\mathcal {O}})$ such that $\pi (\phi )=T$. Notice that in Algorithm 1 we employ both the *return* and the *yield* statements for the output, the difference being that the latter does not halt the algorithm.

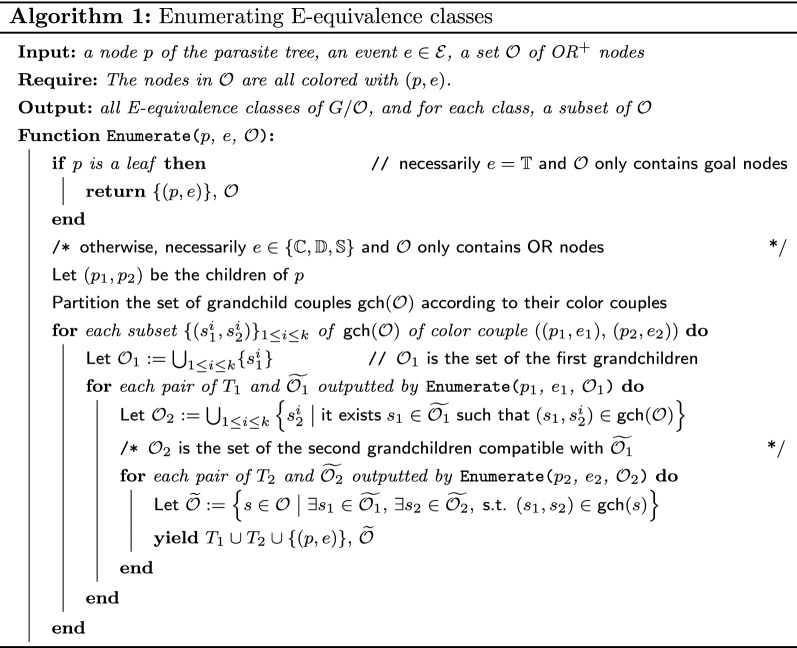



Before the proof of correctness, let us recall some important notations. For a subgraph $G/{\mathcal {O}}$ of the reconciliation graph *G*, a solution subtree is denoted by $\phi \in {\mathcal {T}}(G/{\mathcal {O}})$. The root $\text {OR}^+$ node of a solution subtree $\phi$ is denoted by $r(\phi )$. If the root node $r(\phi )$ is labeled by (*p*, *h*, *e*), the solution subtree $\phi$ is interpreted as an optimal reconciliation between the parasite subtree $P|_p$ and the host tree *H* such that *p* is mapped to *h* and the event of *p* is *e* (for short, we say that $\phi$ is a reconciliation of *P*|*p*). We will use interchangeably the terms *solution subtree* and *reconciliation*, and we will represent a reconciliation $\phi$ as a set of triples.

#### Lemma 13

*In Algorithm 1*, Enumerate(*p*, *e*, ${\mathcal {O}}$) *outputs all E-equivalence classes in* $\pi ({\mathcal {T}}(G/{\mathcal {O}}))$ exactly once, and for each outputted pair of *T* and $\widetilde{{\mathcal {O}}}$, we have $\widetilde{{\mathcal {O}}}=\bigcup _{\phi }\,\{r(\phi )\mid \pi (\phi )=T,\,\phi \in {\mathcal {T}}(G/{\mathcal {O}})\}$.

#### Proof

The proof is by induction on the height $h_p$ of the $P|_p$. We use the fact that the pre-condition in the *Require* statement in Algorithm 1 is true for all recursive calls of Enumerate (easy induction). When $h_p=0$, *p* is a leaf and $\{(p,\sigma (p),{\mathbb {T}})\}$ is the only reconciliation in ${\mathcal {T}}(G/{\mathcal {O}})$, therefore, $\{(p,e)\}$ is the only E-equivalence class. The outputted set ${\mathcal {O}}$ contains in this case the unique goal node of *G* labeled by $(p,\sigma (p),{\mathbb {T}})$. Now we assume $h_p>0$.

*(First direction)* Consider a fixed pair of $T:=T_1\cup T_2\cup \{(p,e)\}$ and $\widetilde{{\mathcal {O}}}$ outputted at Line 16, and take a node *s* in $\widetilde{{\mathcal {O}}}$. We show that there exists a reconciliation $\phi \in {\mathcal {T}}(G/{\mathcal {O}})$ such that $s=r(\phi )$ and $\pi (\phi )=T$ (i.e. *T* is a valid E-equivalence class). By the induction hypotheses, $T_1$ is an E-equivalence class so there exists a reconciliation $\phi _1$ of $P|_{p_1}$ such that $\pi (\phi _1)=T_1$. Let $s_1:=r(\phi _1)$. Take a node $s_2\in {\mathcal {O}}_2$ such that $(s_1,s_2)\in \textsf {gch}(s)$. By the induction hypotheses, there exists a reconciliation $\phi _2$ of $P|_{p_2}$ such that $r(\phi _2)=s_2$ and $\pi (\phi _2)=T_2$. Define $\phi :=\phi _1\cup \phi _2\cup \{(p,h,e)\}$, where (*p*, *h*, *e*) is the label of *s*. Then $\phi$ is a valid reconciliation in ${\mathcal {T}}(G/{\mathcal {O}})$ (notice that $\phi$ is a solution subtree of $G/{\mathcal {O}}$ if and only if $(s_1,s_2)\in \textsf {gch}(s)$), and satisfies $\pi (\phi )=T$.

*(Second direction)* Consider an E-equivalence class $T\in \pi ({\mathcal {T}}(G/{\mathcal {O}}))$, and take a reconciliation $\phi \in {\mathcal {T}}(G/{\mathcal {O}})$ such that $\pi (\phi )=T$. We show that *T* is outputted exactly once at Line 16 together with a set $\widetilde{{\mathcal {O}}}$ containing the root node of $\phi$. Assume that the root node $s:=r(\phi )$ is labeled with the triple (*p*, *h*, *e*), then $\phi$ can be uniquely written as the union $\phi _1\cup \phi _2\cup \{(p,h,e)\}$ where $\phi _1$ and $\phi _2$ are respectively reconciliations of $P|_{p_1}$ and $P|_{p_2}$. Furthermore, *T* can be uniquely written as the union $T_1\cup T_2\cup \{(p,e)\}$ where $T_1=\pi (\phi _1)$ and $T_2=\pi (\phi _2)$. Notice that $T_1$ and $T_2$ do not depend on the choice of $\phi$; for *T* to be outputted exactly once, it suffices to show that each of $T_1$ and $T_2$ is outputted exactly once. For $i=1,2$, let $s_i:=r(\phi _i)$ and let $(p_i,e_i)$ be the color of $s_i$. At Line 10, we only need to consider the iteration corresponding to the color couple $((p_1,e_1),\,(p_2,e_2))$, as no other iteration can output $T_1$ or $T_2$ from a recursive call. Since $s_1\in {\mathcal {O}}_1$ and $\phi _1\in {\mathcal {T}}(G/{\mathcal {O}}_1)$, by the induction hypotheses, $T_1$ is outputted exactly once in Line 12 together with a set $\widetilde{{\mathcal {O}}_1}$ containing $s_1$. For this pair of $T_1$ and $\widetilde{{\mathcal {O}}_1}$, the set ${\mathcal {O}}_2$ computed at Line 13 contains the node $s_2$. Hence, by applying again the induction hypotheses to $\phi _2\in {\mathcal {T}}(G/{\mathcal {O}}_2)$, $T_2$ is outputted exactly once in Line 14 together with $\widetilde{{\mathcal {O}}_2}$ containing $s_2$. It remains to check that the set ${\mathcal {O}}$ outputted together with *T* does contain the node *s*. As $s_i\in \widetilde{{\mathcal {O}}_i}$ for $i=1,2$, this is straightforward from the computation of ${\mathcal {O}}$. $\square$

#### Theorem 14

Using Algorithm 1, the E-equivalence classes of a reconciliation graph can be enumerated in $O(mn^2)$ time delay, where $m=|V(P)|$ and $n=|V(H)|$.

#### Proof

To obtain all E-equivalence classes $\pi ({\mathcal {T}}\,(G))$, it suffices to first partition the set of start nodes of the reconciliation graph according to their colors, then, for each subset ${\mathcal {O}}_i$ of start nodes of color (*p*, *e*), make one call of Enumerate (*p*, *e*, ${\mathcal {O}}$). By Lemma 13, we output every E-equivalence class of ${\mathcal {T}}\,(G/{\mathcal {O}})$ exactly once. Since any E-equivalence class of ${\mathcal {T}}\,(G)$ is an E-equivalence class of ${\mathcal {T}}\,(G/{\mathcal {O}}_k)$ for a unique *k*, we output every E-equivalence class of ${\mathcal {T}}\,(G)$ exactly once.

For the complexity, consider the recursion tree formed by the recursive calls of Enumerate. Notice that each node *p* of the parasite tree corresponds to exactly one recursive call, the size of the recursion tree is thus *O*(*m*). In each recursive call, the partitioning of $\textsf {gch}({\mathcal {O}})$ and the computation of the sets ${\mathcal {O}}_1$, ${\mathcal {O}}_2$, and $\widetilde{{\mathcal {O}}}$ can all be done in time linear in the size of $\textsf {gch}({\mathcal {O}})$, which is $O(n^2)$. Therefore, $O(mn^2)$ time is needed in the worst case between outputting two E-equivalence classes. $\square$

### CD-equivalence class enumeration

For the CD-equivalence relation, the problems of enumerating the equivalence classes and studying one particular equivalence class can be solved using the exact same method as for the E-equivalence relation. One only needs to adapt the Definition 12 of the color of an $\text {OR}^+$ node. Instead of the couple (*p*, *e*), the color of an $\text {OR}^+$ node labeled by $(p,h,e)\in U$ is now a triple: the triple (*p*, *h*, *e*) for $e\ne {\mathbb {S}}$, or, when $e={\mathbb {S}}$, the triple $(p,?,{\mathbb {S}})$ (see Definition 11).

## Experimental results

To evaluate the usefulness of the equivalence classes in practice, we obtained 20 real datasets from the literature. The choice of the datasets was motivated by the goal of covering many different situations (such as different sizes of the trees), different contexts (such as the genes/species one that has been shown to be very closely related to the hosts/parasites context, see for instance [34, 35]), different topologies, etc. We also chose five cost vectors $\vec {c}:=\left( c({\mathbb {C}}),\,c({\mathbb {D}}),\,c({\mathbb {S}}),\,c({\mathbb {L}})\right)$ from the literature, namely $(- 1, 1, 1,1)$ (maximizing the cospeciation), (0, 1, 1, 1) (minimizing the events that lead to incongruencies between the tree topologies), $(0, 1, 2, 1),\,(0, 2, 3, 1)$ (host-switches are more penalized), and (0, 1, 1, 0) which is a vector chosen only for theoretical purposes and does not penalize cospeciations and losses.

### Reducing the space of the optimal solutions

**Fig. 3:**
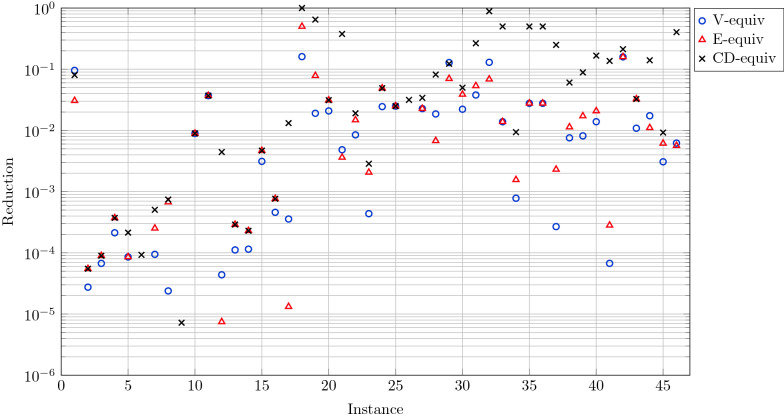
X-axis: All 46 instances (i.e. the pairs of datasets and cost vectors). Y-axis: In logarithmic scale, the Reduction value that is equal to the number of equivalence classes over the total number of reconciliations. For each instance, three points are plotted: the blue circle, the red triangle, and the black X, corresponding respectively to the V-, E-, and CD-equivalence relations. Four points of Reduction values less than $10^{-6}$ are omitted

The goal of the first set of experiments is to check that when the number of all optimal reconciliations is large, the number of equivalence classes is significantly smaller. To this end, we ran the algorithm on all the datasets with all the five cost vectors, and computed the number of optimal solutions and the number of equivalence classes. For each instance (i.e. dataset and cost vector) having at least 50 optimal reconciliations, we computed for each equivalence relation a value that we called *Reduction* and which is equal to the number of equivalence classes over the number of optimal reconciliations. In Fig. 3, each *x* coordinate corresponds to an instance; for each instance we plotted three points that correspond to the Reduction values for the three equivalence relations. One can observe that the Reduction values of the V- and the E-equivalence relations (blue circles and red triangles) are almost all below the value of 0.1. In other words, for these two definitions of equivalence, one can strongly hope for at least a ten-fold decrease, and in some cases for a thousand-fold decrease in the number of reconciliations that need to be analyzed. As expected, the V- and the E-equivalence relations are the ones that usually lead to a small number of equivalence classes, while the CD-equivalence relation may lead to a larger number of classes, sometimes close to the optimal reconciliations (Reduction close to 1).

### The utility of equivalence classes enumeration in the analysis of real datasets

We show now that the equivalence classes not only allow us to reduce the number of reconciliations to consider, but also provide useful information about the set of optimal reconciliations. In particular, we will see that even when the number of optimal reconciliations are too large for exhaustive enumeration, the number of event vectors (V-equivalence classes) can still remain small, and there can be already much biological insight to be gained from the event vectors alone.

To illustrate the utility of our algorithms, we focus on two real datasets among the ones used in the previous experiment. The first is the FD dataset which consists in a host tree of 20 taxa corresponding to species of fish and a tree of their parasites *Dactylogyrus* of 51 taxa [36, 37]. The second is the WOLB dataset representing the *Wolbachia* genus and the various arthropods that host them [38, 39]. This dataset was selected because of its size: the trees have each 387 leaves. In Table 1, we present the detailed results obtained for these datasets and the five cost vectors.

**Table 1:** Experimental results for the FD and *Wolbachia* dataset and for each cost vector

Dataset	|*L*(*H*)|	|*L*(*S*)|	Cost vector	${|{\mathcal {R}}|}$	${|\text {V}_\text {eq}|}$	$|\text {E}_\text {eq}|$	$|\text {CD}_\text {eq}|$
FD [36, 37]	20	51	(−1. 1, 1, 1)	944	8	14	18
			(0, 1, 1, 1)	25184	11	52	72
			(0, 1, 2, 1)	408	10	20	20
			(0, 2, 3, 1)	80	2	2	2
			(0, 1, 1, 0)	$\approx 10^{15}$	2146	54336	$\approx {10^{13}}$
WOLB [38, 39]	387	387	(−1. 1, 1, 1)	$\approx 10^{47}$	10	4080	24192
			(0, 1, 1, 1)	$\approx 10^{48}$	11	40960	76800
			(0, 1, 2, 1)	$\approx 10^{47}$	10	4080	24192
			(0, 2, 3, 1)	$\approx 10^{42}$	7	96	1152
			(0, 1, 1, 0)	$\approx 10^{136}$	–	$\approx 10^{27}$	–

|*L*(*H*)| and |*L*(*S*)| are the number of leaves of the host tree and the parasite tree; $$|{\mathcal {R}}|$$ is the number of optimal reconciliations; $${|\text {V}_\text {eq}|}$$, $$|\text {P}_\text {eq}|$$, and $$|\text {CD}_\text {eq}|$$ are respectively the number of V-, E-, and CD-equivalent classes. The dash indicates that the counting of the equivalence classes did not finish

First notice that even for trees of medium size like in the FD dataset, for the cost vector (0, 1, 1, 1) that is commonly used in the literature, we have 25184 optimal reconciliations which are impossible to be analyzed manually. However, the number of event vectors is only 11; the vectors are: (9, 17, 24, 2), (9, 16, 25, 2), (7, 16, 27, 0), (7, 17, 26, 0), (7, 18, 25, 0), (8, 16, 26, 1), (8, 18, 24, 1), (10, 16, 24, 3), (10, 17, 23, 3), (8, 17, 25, 1), (9, 18, 23, 2). These vectors are all very similar, and can indicate that the parasites have a strong capacity to change hosts (high number of host-switches), while the hosts have a strong capacity to retain their parasites (low number of losses). This is in agreement with what is suggested in the literature that host-switching plays an important role in the evolutionary history of the *Dactylogyrus* species [40]. Moreover, as the number of cospeciations is always lower than the number of duplications, there is evidence that, for this cost vector, the parasites evolve faster than their hosts.

For what concerns the WOLB dataset all the cost vectors lead to a number of optimal reconciliations that is at least $10^{42}$, a number too large for any exhaustive enumeration method. However, in all cases there are only a small number of optimal event vectors (except for the least biologically meaningful cost vector (0, 1, 1, 0)). For the cost vector (0, 2, 3, 1), the seven optimal event vectors are: (102, 0, 284, 36), (103, 0, 283, 39), (104, 0, 282, 42), (105, 0, 281, 45), (106, 0, 280, 48), (107, 0, 279, 51), and (108, 0, 278, 54). From the list of event vectors, one can see that the dataset can be explained by a large number of host-switches and cospeciations, and that there have probably been no duplication.Again this seems in agreement with what is known in the literature as duplications are believed to be a rare event in the evolutionary history of *Wolbachia* whereas host-switches are common [38, 39].

Therefore, by simply considering the equivalence classes one already has an idea of the diversity of the optimal reconciliations. Our approach is thus helpful for drawing conclusions about the co-evolutionary history of this pair of host/parasite association for which few prior analysis methods apply.

### Estimation of event reliability

As there can be a large number of equally optimal reconciliations, the reliability of the predicted evolutionary events may be questioned. It is thus interesting to define support measures that estimate the event reliability (see for example [19]). These measures are mostly based on the idea that in the space of optimal reconciliations, each reconciliation is equally likely and then the support of an event is proportional to the number of optimal reconciliations that confirm it. In this direction, the support of an event can be thought as a rough estimation of the probability of that event in the space of optimal solutions.

The algorithms proposed in this paper allow us to compute these measures efficiently and accurately. Indeed, we can compute not only the equivalence classes but also their size. Once we have the list of event-vectors and the size of each *V*-equivalence class, we have an accurate estimate of the probabilities of the four types of events, assuming that each optimal reconciliation is equally probable. In Table 2 for the WOLB dataset and cost vector (0, 2, 3, 1) we list the *V*-equivalence classes (i.e., the event vectors) together with their size as proportions of the solution space (i.e., the proportion of optimal reconciliations in each *V*-equivalence class among all optimal reconciliations). We can immediately see that $\approx 85\%$ of the optimal reconciliations have $105\pm 1$ cospeciations and it is less probable to find reconciliations with a number of cospeciations far from 105.

**Table 2:** The *V*-equivalence classes for the WOLB dataset, cost vector (0, 2, 3, 1) and their size, as proportions of the solution space, sorted in the decreasing order of the size

Event vector	Proportion of the solution space (%)
(105, 0, 281, 45)	$36.5425$
(106, 0, 280, 48)	$29.5704$
(104, 0, 282, 42)	$18.7570$
(107, 0, 279, 51)	$10.5588$
(103, 0, 283, 39)	$3.1628$
(108, 0, 278, 54)	$1.3807$
(102, 0, 284, 36)	$0.0277$

We could also extend this argument to the *E*-equivalence classes. Recall that an *E*-equivalence class can be viewed as a labeling of the nodes of the parasite tree with an event type. In this case, the support of the pair (node of the parasite tree, event) is proportional to the number of optimal reconciliations that confirm it. In particular, it is interesting to identify the nodes of the parasite tree that are labeled by the same event in *all* the *E*-equivalence classes. This may seem a strong requirement but in practice, for the datasets we analyzed, this number is significant. For the WOLB dataset, only 15 nodes are assigned to different event types, in other words, all the other 371 internal nodes receive a consistent event type across the entire solution space. This means that we have further confirmed that the diversity of the solution space is low: not only the event vectors are similar, the distributions of the events on the nodes of the parasite tree are also similar.

Finally, the algorithm is quite efficient in practice, as for example for the cost vector $(-1,1,1,1)$, to enumerate all the optimal event vectors, it took around 8 minutes for the dataset of *Wolbachia* and their arthropod hosts on a single thread of the Intel Core i5-3380M CPU. The enumeration of equivalence classes, together with other features such as the visualization of the E- and the CD-equivalence classes, is freely available in the software Capybara; more information can be found in [32].

## Discussion

### Comparison with eMPRess

eMPRess [10, 13] is a tool that includes the possibility for the user to cluster the space of optimal solutions using agglomerative hierarchical clustering. The user can define the desired final number of clusters and a lower bound for the initial number of clusters (the actual initial number depends on the structure of the reconciliation graph, and can be much larger than the chosen lower bound). Then, pairs of clusters are merged using a linkage criterion until the desired number of clusters is obtained. The authors consider two different linkage criteria: (i) minimizing the average distance between the solutions within each cluster with respect to a given distance metric (the symmetric distance or the path distance), (ii) maximizing the average event support in each cluster.

As already mentioned in the introduction, the approach of eMPRess is fundamentally different from the one we propose. We believe that it is interesting to remark some of the differences between the two methods that the user should keep in mind when applying one method or the other.

It is important to notice that the results obtained with our algorithm and with eMPRess can be very different. Two solutions that may be considered equivalent may have a large symmetric or path distance. Indeed, the symmetric distance between two reconciliations is defined as the number of associations that are found in one reconciliation or the other but not in both. Inside an E-equivalence class, even though the type of the events is consistent among the reconciliations, all the associations can potentially be different, so the symmetric distance can take the largest possible value. Moreover, when using the event support criterion, it is important to keep in mind that within a cluster, by construction, the more ancestral events are more supported than the more recent events. While this may be biologically motivated, it is a bias that we may not want in some datasets.

These differences are also seen in practice as we applied eMPRess to some of the datasets used in the previous section, requiring that the number of final clusters is the same (or slightly larger) than the number of equivalence classes that we have found for that dataset. By analyzing the median reconciliations of the final clusters, we saw that, even for the V-equivalence relation (which is among those most analyzed in practical studies), some classes are not represented.

Finally, the worst case running time of the clustering method of eMPRess depends quadratically on the initial number of clusters and the time can be a limitation in practice. When we applied it to the *Wolbachia* dataset with the default cost vector (0, 2, 3, 1) and the symmetric distance criterion, by starting with 336 initial clusters (level $L=6$ in [13]) and choosing 10 as the final number of clusters, the software did not finish within 24 h.

## Conclusion

In this paper, we proposed a method that lists representative reconciliations from the (often huge) space of optimal solutions. To this purpose, we first defined when two reconciliations can be considered equivalent and then we provided efficient algorithms that output in polynomial delay only one reconciliation from each equivalence class. We proposed three different biologically motivated equivalence relations. We applied our algorithms to real datasets and showed that we were able to analyze the space of optimal reconciliations even in cases when the latter has a huge size (e.g. $10^{42}$). As a future direction, we plan to extend our algorithms to other definitions of equivalence for reconciliations.
